# Rechecking the Centrality-Lethality Rule in the Scope of Protein Subcellular Localization Interaction Networks

**DOI:** 10.1371/journal.pone.0130743

**Published:** 2015-06-26

**Authors:** Xiaoqing Peng, Jianxin Wang, Jun Wang, Fang-Xiang Wu, Yi Pan

**Affiliations:** 1 School of Information Science and Engineering, Central South University, Changsha, Hunan, 410083, China; 2 Department of Molecular Physiology & Biophysics, Baylor College of Medicine, Houston, Texas 77030, USA; 3 Department of Mechanical Engineering and Division of Biomedical Engineering, University of Saskatchewan, Saskatoon, Canada; 4 Department of Computer Science, Georgia State University, Atlanta, GA 30302-4110, USA; Umeå University, SWEDEN

## Abstract

Essential proteins are indispensable for living organisms to maintain life activities and play important roles in the studies of pathology, synthetic biology, and drug design. Therefore, besides experiment methods, many computational methods are proposed to identify essential proteins. Based on the centrality-lethality rule, various centrality methods are employed to predict essential proteins in a Protein-protein Interaction Network (PIN). However, neglecting the temporal and spatial features of protein-protein interactions, the centrality scores calculated by centrality methods are not effective enough for measuring the essentiality of proteins in a PIN. Moreover, many methods, which overfit with the features of essential proteins for one species, may perform poor for other species. In this paper, we demonstrate that the centrality-lethality rule also exists in Protein Subcellular Localization Interaction Networks (PSLINs). To do this, a method based on Localization Specificity for Essential protein Detection (LSED), was proposed, which can be combined with any centrality method for calculating the improved centrality scores by taking into consideration PSLINs in which proteins play their roles. In this study, LSED was combined with eight centrality methods separately to calculate Localization-specific Centrality Scores (*LCS*s) for proteins based on the PSLINs of four species (*Saccharomyces cerevisiae*, *Homo sapiens*, *Mus musculus* and *Drosophila melanogaster*). Compared to the proteins with high centrality scores measured from the global PINs, more proteins with high *LCS*s measured from PSLINs are essential. It indicates that proteins with high *LCS*s measured from PSLINs are more likely to be essential and the performance of centrality methods can be improved by LSED. Furthermore, LSED provides a wide applicable prediction model to identify essential proteins for different species.

## Introduction

Different proteins play different roles and have different degrees of importance in biological activities of organisms. There is a kind of proteins, named essential proteins, which are needed by living organisms to maintain life activities, and the organism cannot survive or grow without them [1, 2]. The study of essential proteins can facilitate other researches. For example, determining a minimal set of essential genes for a simplest free-living organism is fundamental in synthetic biology [3, 4]. In the field of the resistance to antibiotics and toxicity, studying the essential proteins in bacteria and viruses can help design new antimicrobial drugs, since the bacteria and viruses may die from removing, interrupting or obstructing their essential proteins [5].

Essential proteins can be identified by biological experiments, such as single gene knockout [6], RNA interference [7] and conditional knockout [8]. However, these experiments are both time consuming and inefficient, and can only be applied to a few species. Thus, it is appealing to develop highly reliable and efficient computational methods to identify essential proteins.

Fast growth in the amount of available Protein-Protein Interactions (PPIs) has provided unprecedented opportunities for detecting essential proteins at the network level. In *Saccharomyces cerevisiae*, proteins with high degrees in a Protein-protein Interaction Network (PIN) are more likely to be encoded by essential genes and thus more likely to be essential proteins [9]. From the perspective of topology, highly connected proteins can maintain the basic structures of PIN, and the whole PIN will collapse if these proteins are removed. This phenomenon is called the centrality-lethality rule in biological networks [10]. Thus some centrality methods have been used to measure the essentiality of proteins, for example, Degree Centrality (DC) [9], Betweenness Centrality (BC) [11], Closeness Centrality (CC) [12], Subgraph Centrality (SC) [13], Eigenvector Centrality (EC) [14], and Information Centrality (IC) [15]. Later on, some other centrality measures have been proposed by looking into the topology properties of essential proteins’ neighborhoods. By investigating the essentiality of proteins and their neighbors in *Saccharomyces cerevisiae* PIN, Lin *et al*. [16] proposed maximum neighborhood component and density of maximum neighborhood component algorithms to identify essential proteins. Li *et al*. [17] found that the neighbors of non-essential hubs seldom interact with each other, thus they proposed a method based on local average connectivity. Wang *et al*. [18] proposed a centrality measure based on edge clustering coefficient, named NC.

However, the available PPI data is incomplete and contains false-positives, which will affect the accuracy of essential protein prediction methods that are solely based on topology. Much information provided by the high-throughput experiments can help reduce the influence of false-positives and capture the characteristics of essential proteins from other angles. Thus a new trend to improve the essential protein identification is to integrate other information with PINs. Based on the combination of logistic regression-based model and function similarity, Li *et al*. [19] proposed a weighting method to evaluate the confidence of each PPI, and the accuracies of nine centrality measures in the weighted network were improved. Luo *et al*. [20] utilized Gene Ontology to obtain a weighted network, and calculated local topological characteristics of proteins in the weighted networks to identify essential proteins. Recently, the relationship between protein essentiality and their cluster property have been considered when identifying essential proteins. Elena *et al*. [21] reexamined the connection between the network topology and essentiality. As a result, they observed that the majority of hubs are essential due to their involvement in Essential Complex Biological Modules, a group of densely connected proteins with shared biological functions enriched in essential proteins. Based on this observation, Ren *et al*. [22] integrated the topology of PINs and protein complexes information to predict essential proteins. Li *et al*. [23] proposed a new prediction method based on Pearson correlation coefficient and edge clustering coefficient, named PeC, and Tang *et al*. [24] proposed a Weighted Degree Centrality method (WDC). Both PeC and WDC integrate network topology with gene expression profiles. Considering that essential proteins tend to be conservative, Peng *et al*. [25] proposed an iteration method (ION) for predicting essential proteins by integrating the protein orthology information [26] with PINs.

In addition, some machine learning methods have been developed for identifying essential proteins. For example, Acencio *et al*. [27] constructed a decision tree-based meta-classifier and trained it on datasets with the integration of network topological features, cellular localization and biological process, to explore essential proteins. Zhong *et al*. [28] proposed a GEP-based method to predict essential proteins by combining biological features, classical topological features, and other composed features computed by the PeC, WDC and ION methods. However, when applying to other species, the performances of these supervised machine learning methods may be affected by the differences between the training species and the prediction species [29]. All above methods try to improve the essential protein identification from different angles. However, due to the incompleteness and the dynamics of PPIs, it still lacks efficient methods to identify essential proteins accurately for different species.

In the network-based methods aforementioned, the PINs are constituted by all PPIs available at the moment which may take place in different subcellular localizations (denoted as global PINs). However, proteins must be localized at their appropriate subcellular compartments to perform their desired functions [30–34], and PPIs can take place only when proteins are in the same subcellular localization [31, 35]. In this paper, we demonstrated that the centrality-lethality rule also exists in Protein Subcellular Localization Interaction Networks (PSLINs), which are constituted by proteins and their PPIs in the same subcellular localization. A number of proteins and essential proteins from different PSLINs are significantly different. This paper proposes a method based on Localization Specificity for Essential protein Detection (LSED), which can be combined with any centrality method to calculate Localization-specific Centrality Scores (*LCS*s) for proteins based on PSLINs. LSED combined with a certain centrality method XC is denoted as LSED-XC, in which the centrality method XC is applied to each PSLIN to calculate centrality scores of proteins. Based on the centrality scores from different PSLINs, a Localization-specific Centrality Score (*LCS*) is calculated for each protein and the localization-specific essential proteins are largely explored. The results show that, compared to the proteins with high centrality scores measured from the global PINs, more proteins with high *LCS*s measured from PSLINs are essential. It indicates that compared with the centrality method XC applied to the global PINs, LSED-XC can improve the accuracy of centrality methods for essential protein predictions of different species.

## Materials and Methods

### Materials

In this study, the prediction methods were applied to four species (*Saccharomyces cerevisiae*, *Homo sapiens*, *Mus musculus*, and *Drosophilamelanogaster*) for essential protein identification. The PINs of four species were downloaded from Biogrid database [36]. All these PINs are the mixtures of PPIs from different subcellular localizations, and are considered as the global PINs. Their statistics are summarized in Table 1. For each species, the known essential proteins were extracted from DEG [2] and used as the benchmark set to evaluate the essential protein predictions.

**Table 1:** Statistics of proteins, PPIs, essential proteins, and proteins annotated by 11 labeled compartments in the global PIN of each species.

Species	# *Protein*	# *PPI*	# *Essential protein*	# *Annotated protein*
*Saccharomyces cerevisiae*	6,304	81,614	1,098	4,317
*Homo sapiens*	16,275	143,611	2,342	12,905
*Mus musculus*	6,582	17,460	1,304	5,523
*Drosophila melanogaster*	8,020	36,334	255	2,910

#Protein, #PPI, #*Essential protein*, and #*Annoted Protein* denote the numbers of proteins, PPIs, essential proteins and proteins annotated by 11 labeled compartments in the global PIN of each species, respectively.

The localization information of proteins in COMPARTMENTS database [37] was used in this study. The subcellular localizations (or compartments) in a cell are generally classified into the following 12 categories: 1) *Chloroplast*, 2) *Endoplasmic*, 3) *Cytoskeleton*, 4) *Golgi*, 5) *Cytosol*, 6) *Lysosome*(or *Vacuole*), 7) *Mitochondrion*, 8) *Endosome*, 9) *Plasma*, 10) *Nucleus*, 11) *Peroxisome* and 12) *Extracellular*, where *Chloroplast* only exists in plant cells [38]. These labeled compartments are used to annotate the localization of proteins based on the supporting evidence. The number of proteins annotated by these compartments in the global PIN of each species is listed in Table 1.

### The Framework of LSED

**Fig 1:**
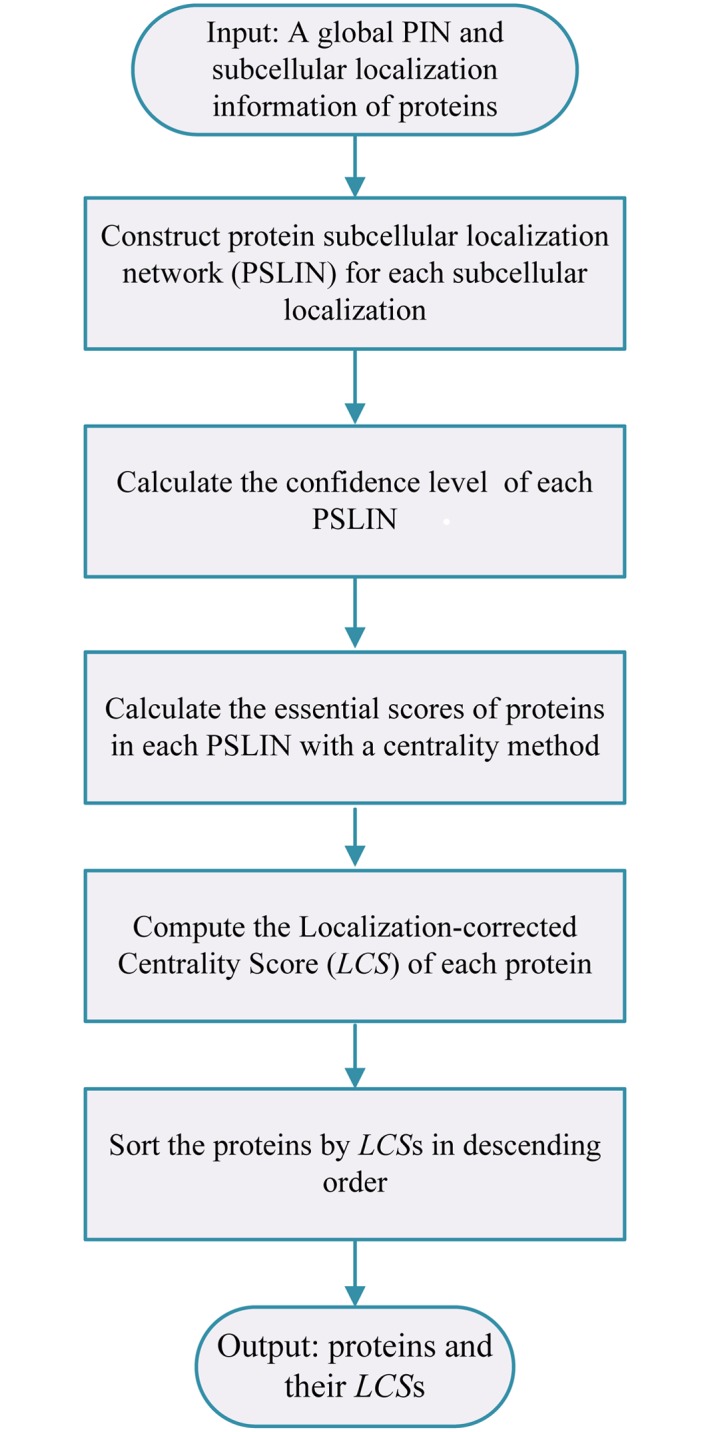
The schematic of LSED method.

The LSED method mainly contains four steps, as shown in Fig 1. Given a global PIN and subcellular localization information of proteins, firstly, a PSLIN was constructed for each subcellular localization. Secondly, the confidence level of each PSLIN is calculated according to the size of the PSLIN. In the third step, a centrality method is applied to each PSLIN for calculating the centrality scores of proteins in the PSLIN. Then the *LCS* of each protein is calculated based on its centrality scores in different PSLINs and the confidence levels of these PSLINs. Finally, the proteins are sorted by their *LCS*s in descending order. The details of each step will be discussed in the following subsections.

### Construction of PSLINs

**Fig 2:**
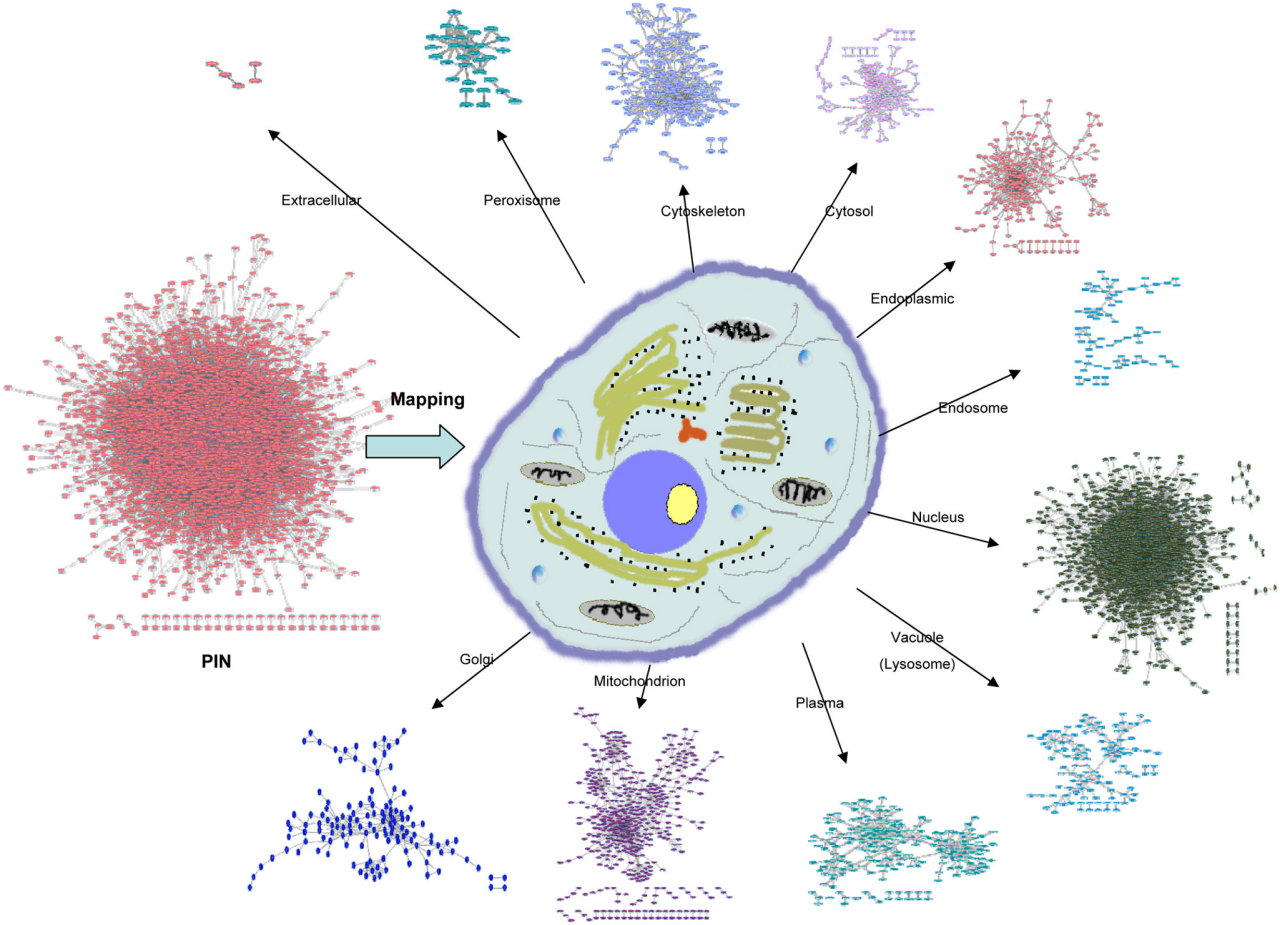
Construction of PSLIN. A network in the left represents a global PIN. A eukaryotic cell in the center can be divided into 11 compartments, *Endoplasmic*, *Cytoskeleton*, *Golgi*, *Cytosol*, *Lysosome*(or *Vacuole*), *Mitochondrion*, *Endosome*, *Plasma*, *Nucleus*, *Peroxisome* and *Extracellular*, where *Lysosome* only exists in animal cells. For each compartment, a PSLIN of this compartment is constituted by the proteins localized in this compartment and their interactions. With the subcellular localization information of proteins, the PSLINs can be generated by mapping the global PIN to each compartment separately.

The PPIs in a global PIN are identified from various in vitro conditions without knowledge of subcellular localization where they take place. Proteins must be localized to the correct compartments [30–34] and the interacting protein pairs should be in the same subcellular localization [31, 35]. Thus, a global PIN can be divided into a number of PSLINs based on subcellular localizations. A eukaryotic cell can be divided into 11 compartments, *Endoplasmic*, *Cytoskeleton*, *Golgi*, *Cytosol*, *Lysosome*(or *Vacuole*), *Mitochondrion*, *Endosome*, *Plasma*, *Nucleus*, *Peroxisome* and *Extracellular*, where *Lysosome* only exists in animal cells. The PSLIN of each compartment is constituted by the proteins localized in this compartment and their interactions. If a protein is annotated by multiple subcellular localizations, it will appear in multiple PSLINs. Let *G* = (*V*, *E*) denote the global PIN, and *Loc*(*i*) denote the set of proteins in compartment *i*. The PSLIN of compartment *i* can be denoted as *S*_*i*_ = (*V*_*i*_, *E*_*i*_), where *V*_*i*_ = {*v*∣*v* ∈ *V*∩*Loc*(*i*)}, and *E*_*i*_ = {*e*(*u*, *v*)∣*e*(*u*, *v*) ∈ *E*, *u* ∈ *V*_*i*_, *v* ∈ *V*_*i*_}. As shown in Fig 2, with the subcellular localization information of proteins, the PSLINs can be generated by mapping the global PIN to each compartment separately. For more information about the PSLINs of *Saccharomyces cerevisiae*, *Homo sapiens*, *Mus musculus*, and *Drosophila melanogaster*, see S1 Dataset, S2 Dataset, S3 Dataset and S4 Dataset in the Supporting Information files of this paper.

### Localization-specific Centrality Score

A protein may appear in several different PSLINs, and it will have several centrality scores calculated from these PSLINs. A *LCS* is calculated to measure the essentiality of proteins. The *LCS* of a protein depends on its centrality scores from various PSLINs and the reliability of these centrality scores. In this study, the reliability of the centrality score from a PSLIN is measured by the confidence level of the PSLIN. The size of a network is defined as the number of proteins in the network. Intuitively, the larger its size, the higher confidence level of a PSLIN should be (see more explanations in Discussion). Let *S*_*Max*_ denote the PSLIN with the largest size (containing the largest number of proteins), ∣*∣ denotes the size of the PSLIN *. Therefore, in this study, the confidence level of a PSLIN is calculated by the ratio of its size to the largest size of PSLINs as follows.

$$C\left(S_{i}\right)=\frac{|S_{i}|}{|S_{Max}|}$$ (1)

where *S*_*i*_ is the PSLIN of compartment *i*. From the definition, the value of *C*(*S*_*i*_) is in the range of (0, 1]. For more information about the confidence levels of different PSLINs of four species, see S1 Table in the Supporting Information files of this paper.

The details for calculating the *LCS*s of proteins is described Algorithm 1. To calculate the *LCS*s of proteins, firstly, all PSLINs are sorted in descending order according to their confidence levels. Then, the centrality scores of each protein in the sorted PSLINs are calculated. If a protein appears in a PSLIN, its centrality score in the PSLIN is calculated by a centrality method; otherwise, its centrality score is zero. Later on, for each protein, its *LCS* is calculated based on its centrality scores computed from the sorted PSLINs and the confidence levels of these PSLINs. After the *LCS*s of all proteins in the global PIN are calculated, the proteins are sorted in descending order by their *LCS*s.

### Evaluation Metrics

Selecting a certain number of proteins as candidates for essential proteins, the percentage of true essential proteins can be calculated according to the list of known essential proteins. Many centrality methods were evaluated by comparing the percentage/number of essential proteins in the top ranked proteins (the proteins with high centrality scores) [16–18, 25, 39–41]. In this paper, we also adopt this metric to evaluate the prediction accuracy of each method.

**Algorithm 1** The calculation of *LCS*

1: **Input:** The sorted PSLINs set *PS* = {*S*_1_, …, *S*_*i*_, …, *S*_*n*_∣*C*(*S*_*i*−1_) ≥ *C*(*S*_*i*_) ≥ *C*(*S*_*i*+1_)} and the protein set *V*

2: // *n* is the number of PSLINs and *V* denotes all the proteins in the global PIN.

3: **Output:** The *LCS*s of proteins in *V*

4:

5: //Calculate the centrality scores of each protein in PSLINs

6: **for** *i* = 1; *i* ≤ *n*; *i*++ **do**

7:  // Calculate the centrality score *Ess*(*p*, *S*_*i*_) of each protein *p* in PSLIN *S*_*i*_

8:  **for** each protein *p* ∈ *V* **do**

9:   **if** *p* ∈ *S*_*i*_ **then**

10:    *Ess*(*p*, *S*_*i*_) is calculated by a centrality method

11:   **else**

12:    *Ess*(*p*, *S*_*i*_) = 0

13:   **end if**

14:  **end for**

15: **end for**

16:

17: //Calculate the *LCS* of each protein *p*

18: **for** each protein *p* ∈ *V* **do**

19:  *LCS*(*p*) = 0

20:  **for** *i* = 1; *i* ≤ *n*; *i*++ **do**

21:   **if** $Ess\left(p,\;S_{i}\right)⪈LCS(p)$ **then**

22:    *LCS*(*p*) = *LCS*(*p*)+(*Ess*(*p*, *S*_*i*_)-*LCS*(*p*))*C(*S*_*i*_)

23:   **end if**

24:  **end for**

25: **end for**

26: Sort the proteins in descending order by their *LCS*s

27: Output the *LCS*s of proteins in *V*

#### Comparison of the Top Percentages of Ranked Proteins

Assume that proteins are sorted by their centrality scores in descending order. The top 1%, 5%, 10%, 15%, 20%, and 25% of ranked proteins are selected as predicted essential proteins, and then the percentage of true essential proteins correctly identified by each method is compared. In this paper, LSED-XC represents LSED is combined with a certain centrality method XC. XC can be DC, IC, EC, SC, BC, CC, NC, as well as ION, while the corresponding LSED-XC can be LSED-DC, LSED-IC, LSED-EC, LSED-SC, LSED-BC, LSED-CC, LSED-NC, as well as LSED-ION. In the comparison of the enrichment level of essential proteins in the top percentages of ranked proteins, Eqs (2)–(5) are defined to explain the comparison between LSED-XC and XC methods.

Accuracy (*Acc*): given a certain value of *c*, the accuracy of a method *M* in the top *c*% of ranked proteins is defined as the percentage of true essential proteins identified by method *M* in the top *c*% of ranked proteins, calculated according to Eq (2).

$$Acc\left(M,c\right)=\frac{TP}{N}\times 100\%$$ (2)

where *TP* is the number of true essential proteins identified by method *M* in the top *c*% of ranked proteins, and *N* is the number of the top *c*% of ranked proteins.

Improved accuracy (*IAcc*): the improved accuracy of method LSED-XC in the top *c*% of ranked proteins, calculated according to Eq (3), is defined as the improvement of the *Acc* of LSED-XC compared to the *Acc* of the corresponding method XC.

$$IAcc\left(LSED-XC,c\right)=\frac{Acc(LSED-XC,c)-Acc(XC,c)}{Acc(XC,c)}$$ (3)

Average Improved Accuracy(*AIAcc*): the average value of improved accuracy of a method LSED-XC is defined as the average value of the *IAcc* of LSED-XC in different top percentages of ranked proteins, and is calculated according to Eq (4), where *Topcset* is a set of different percentages.

$$AIAcc\left(LSED-XC,Topcset\right)=\frac{\sum_{c\in Topcset}IAcc\left(LSED-XC,c\right)}{\left|Topcset\right|}$$ (4)

where ∣*Topcset*∣ denotes the number of different percentages in *Topcset*.

#### Comparison of the Average Accuracy over Species

To evaluate the prediction accuracy of each method more comprehensively, the average accuracy (*AKAcc*) of a method *M* in the top *c*% of ranked proteins over more than one species is calculated by Eq (5).

$$AKAcc\left(M,c,k\right)=\frac{\sum_{i=1}^{k}Acc_{i}\left(M,c\right)}{k}$$ (5)

where *k* is the number of species, *Acc*_*i*_(*M*, *c*) is the accuracy of *M* in the top *c*% of ranked proteins of species *i*. The higher the *AKAcc* gained by a method, the higher the likelihood of success prediction for different species.

## Results

To recheck the centrality-lethality rule in the scope of PSLINs, we carried out experiments for four species, *Saccharomyces cerevisiae*, *Homo sapiens*, *Mus musculus*, and *Drosophila melanogaster*. In our experiments, seven typical topology-based centrality methods (DC, IC, EC, SC, BC, CC, and NC) and a centrality method integrating with other biological knowledge (ION) were adopted, respectively. LSED was combined with these centrality methods (denoted by LSED-XC) to calculate centrality scores from PSLINs separately. The proteins were sorted by the *LCS*s calculated with LSED-XC in descending order. Specifically, the orthology frequency of each protein in a species needed by ION [25] was calculated among 272 species from INPARANIOD database [26]. In addition, for the sake of comparison, each centrality method was applied to the global PINs independently, and the proteins were sorted by centrality scores in descending order, too. In the following, top 1%–25% means the set 1%, 5%, 10%, 15%, 20%, 25%. For more information about the proteins of each species with *LCS*s calculated by LSED-XC and centrality scores calculated by the corresponding centrality methods, see S1 File, S2 File, S3 File and S4 File in the Supporting Information files of this paper.

### Saccharomyces cerevisiae

**Fig 3:**
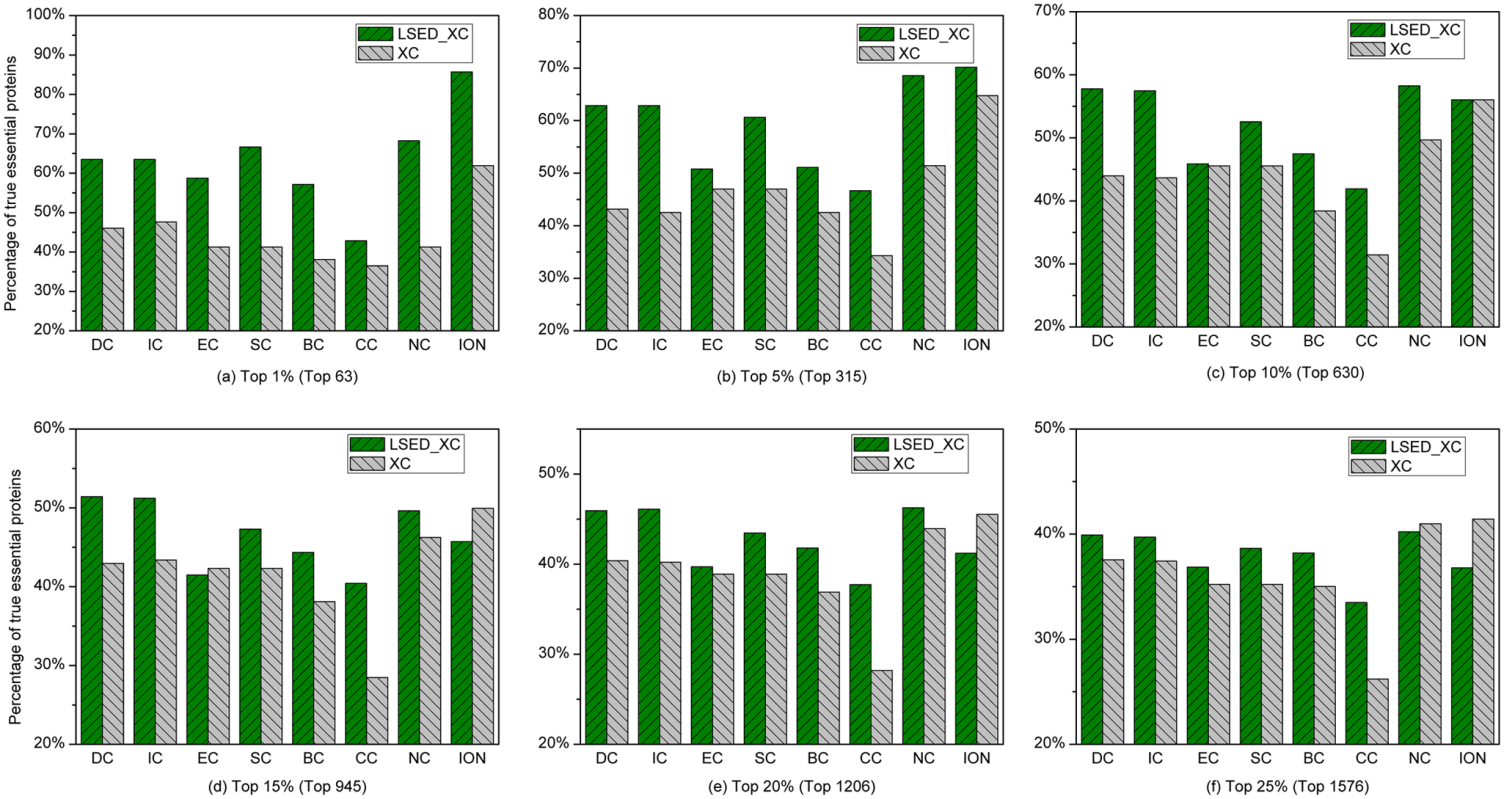
Percentage of top *c*% ranked proteins, identified by LSED-XC methods and XC methods, to be essential proteins of *Saccharomyces cerevisiae*. Eight centrality methods (DC, BC, CC, SC, EC, IC, NC, and ION) were adopted to calculate centrality scores from the global PIN, respectively. LSED was combined with these centrality methods to calculate Localization-specific Centrality Scores from PSLINs separately. In (a)-(f), all the centrality methods are denoted as XC in the legend, and LSED with different XC methods are denoted as LSED-XC in the legend. The proteins are ranked in the descending order based on their Localization-specific Centrality Scores (*LCSs*) and centrality scores computed by LSED-XC methods and XC methods, respectively. Then, top 1%, 5%, 10%, 15%, 20% and 25% of the ranked proteins are selected as candidates for essential proteins. According to the list of known essential proteins, the percentages of true essential proteins were calculated. The figure shows the percentage of true essential proteins identified by each method in each top percentage of ranked proteins. The digits in brackets stand for the number of proteins ranked in each top percentage. For example, since the total number of ranked proteins of *Saccharomyces cerevisiae* is 6,304, the number of proteins ranked in top 1% is about 63 (= 6,304*1%).

Fig 3 shows the percentage of true essential proteins identified by LSED-XC methods and XC methods in each top percentage of ranked proteins of *Saccharomyces cerevisiae*. For most topology-based centrality methods, we can observe that the percentages of true essential proteins correctly predicted by LSED-XC methods are greatly higher than those of the corresponding XC methods in the top 1%–25% of ranked proteins. The *IAcc* of LSED-XC methods is shown in Table 2. In Table 2, the positive value of *IAcc* gained by LSED-XC method indicates that the *Acc* of the corresponding XC method can be improved by LSED method. LSED-DC, LSED-IC, LSED-EC, LSED-SC, LSED-BC, LSED-CC, and LSED-NC always gain positive values of *IAcc* in the top 1%–25% of ranked proteins of *Saccharomyces cerevisiae*.

**Table 2:** The improved accuracy (*IAcc*) of LSED-XC method compared to the accuracy of the corresponding XC method in the top *c*% of ranked proteins of *Saccharomyces cerevisiae*.

Method	*IAcc*					
	Top 1%	Top 5%	Top 10%	Top 15%	Top 20%	Top 25%
LSED-DC	37.93%	45.59%	31.41%	19.70%	13.63%	6.25%
LSED-IC	25.00%	32.32%	24.03%	15.29%	12.76%	5.75%
LSED-EC	42.31%	8.11%	0.70%	-2.00%	3.73%	4.68%
LSED-SC	61.54%	29.05%	15.33%	11.75%	10.79%	9.73%
LSED-BC	50.00%	20.15%	23.55%	16.39%	12.90%	9.06%
LSED-CC	17.39%	36.11%	33.33%	42.01%	30.68%	27.85%
LSED-NC	65.38%	33.33%	17.25%	7.32%	1.25%	-1.86%
LSED-ION	38.46%	8.33%	0.00%	-8.47%	-10.07%	-11.18%

By integrating with orthology information, the percentages of true essential proteins correctly identified by ION are much greater than those of other XC methods in the top 1%–25% of ranked proteins. However, the *Acc* of XC methods can be greatly improved when considering protein subcellular localization. In the top 1% of ranked proteins, LSED-DC, LSED-IC, LSED-SC, LSED-NC, and LSED-ION outperform ION; In the top 5% of ranked proteins, the *Acc*s of LSED-NC and LSED-ION are greater than that of ION; In the top 10%-20% of ranked proteins, more true essential proteins are identified by LSED-DC and LSED-IC methods. It demonstrates that both protein sublocalization information and orthology information are helpful for identifying essential proteins in *Saccharomyces cerevisiae*. For more information about the true essential proteins in the top percentages of proteins ranked by LSED-XC methods and XC methods in *Saccharomyces cerevisiae*, see S2 Table in the Supporting Information files of this paper.

### Homo sapiens

**Fig 4:**
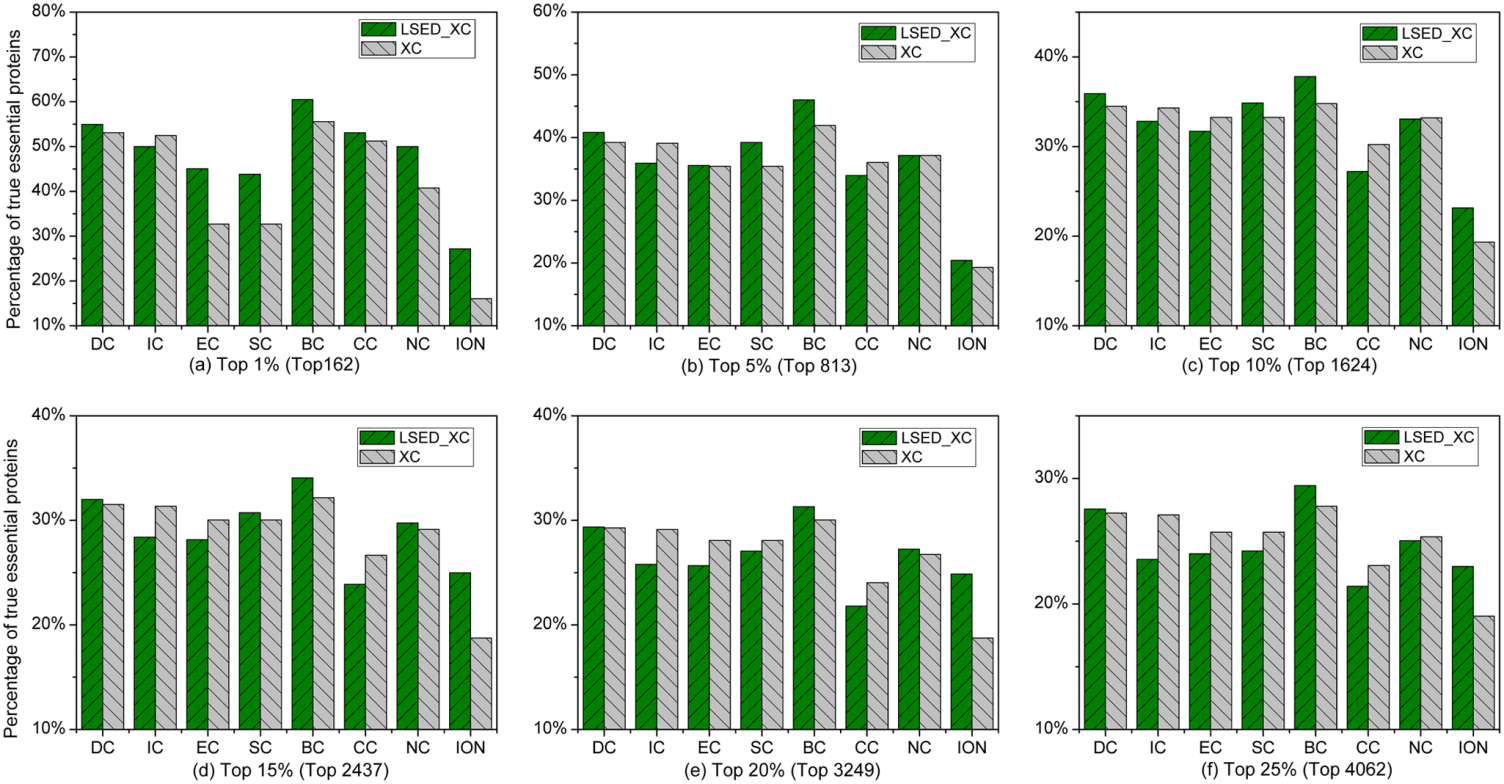
Percentage of top *c*% ranked proteins, identified by LSED-XC methods and XC methods, to be essential proteins of *Homo sapiens*. Eight centrality methods (DC, BC, CC, SC, EC, IC, NC, and ION) were adopted to calculate centrality scores from the global PIN, respectively. LSED was combined with these centrality methods to calculate Localization-specific Centrality Scores from PSLINs separately. In (a)-(f), all the centrality methods are denoted as XC in the legend, and LSED with different XC methods are denoted as LSED-XC in the legend. The proteins are ranked in the descending order based on their Localization-specific Centrality Scores (*LCSs*) and centrality scores computed by LSED-XC methods and XC methods, respectively. Then, top 1%, 5%, 10%, 15%, 20% and 25% of the ranked proteins are selected as candidates for essential proteins. According to the list of known essential proteins, the percentages of true essential proteins were calculated. The figure shows the percentage of true essential proteins identified by each method in each top percentage of ranked proteins. The digits in brackets stand for the number of proteins ranked in each top percentage. For example, the total number of ranked proteins of Homo sapiens is 16,275, thus the number of proteins ranked in top 1% is about 162 (= 16,275*1%).

In Fig 4, the percentage of true essential proteins correctly predicted by LSED-XC methods is compared to that by XC methods in each top percentage of ranked proteins of *Homo sapiens*. In the top 1%–25% of ranked proteins, the percentages of top *c*% ranked proteins, identified by DC and BC, to be essential proteins are higher than those of other XC methods, while LSED-DC and LSED-BC outperform DC and BC, respectively.

From Table 3, we can observe that LSED-DC, LSED-BC, and LSED-ION always gain positive values of *IAcc* in the top 1%–25% of ranked proteins of *Homo sapiens*. Compared with SC, the positive values of *IAcc* gained by LSED-SC are mainly in the top 1–15% of ranked proteins. For EC, CC and NC, the positive values of *IAcc* gained by the corresponding LSED-XC methods are mainly in the top 1% of ranked proteins.

**Table 3:** The improved accuracy (*IAcc*) of LSED-XC method compared to the accuracy of the corresponding XC method in the top *c*% of ranked proteins of *Homo sapiens*.

Method	*IAcc*					
	Top 1%	Top 5%	Top 10%	Top 15%	Top 20%	Top 25%
LSED-DC	3.49%	4.08%	4.11%	1.56%	0.32%	1.17%
LSED-IC	−4.94%	−8.90%	−4.50%	−10.40%	−12.89%	−15.05%
LSED-EC	37.74%	0.35%	−4.63%	−6.28%	−8.55%	−6.70%
LSED-SC	33.96%	10.76%	4.81%	2.32%	−3.62%	−5.84%
LSED-BC	8.89%	9.68%	8.67%	5.87%	4.20%	5.93%
LSED-CC	3.61%	−5.80%	−9.98%	−10.46%	−9.35%	−7.15%
LSED-NC	22.73%	0.00%	−0.37%	2.11%	1.84%	−1.26%
LSED-ION	69.23%	5.73%	19.75%	33.26%	32.68%	20.83%

Compared with the XC methods based on topology, the percentages of true essential proteins correctly identified by ION in top 1%–25% of ranked proteins are quite low. Compared with NC, which is used to initialize the centrality scores in ION, ION predicts less true essential proteins in top 1%–25% of ranked proteins. It seems that the orthology information of *Homo sapiens* proteins used in ION degrades the performance of NC. However, LSED-ION outperforms ION in the top 1%–25% of ranked proteins, which demonstrates the effectiveness of both LSED method and PSLINs. For more information about the true essential proteins in the top percentages of proteins ranked by LSED-XC methods and XC methods in *Homo sapiens*, see S3 Table in the Supporting Information files of this paper.

### Mus musculus

**Fig 5:**
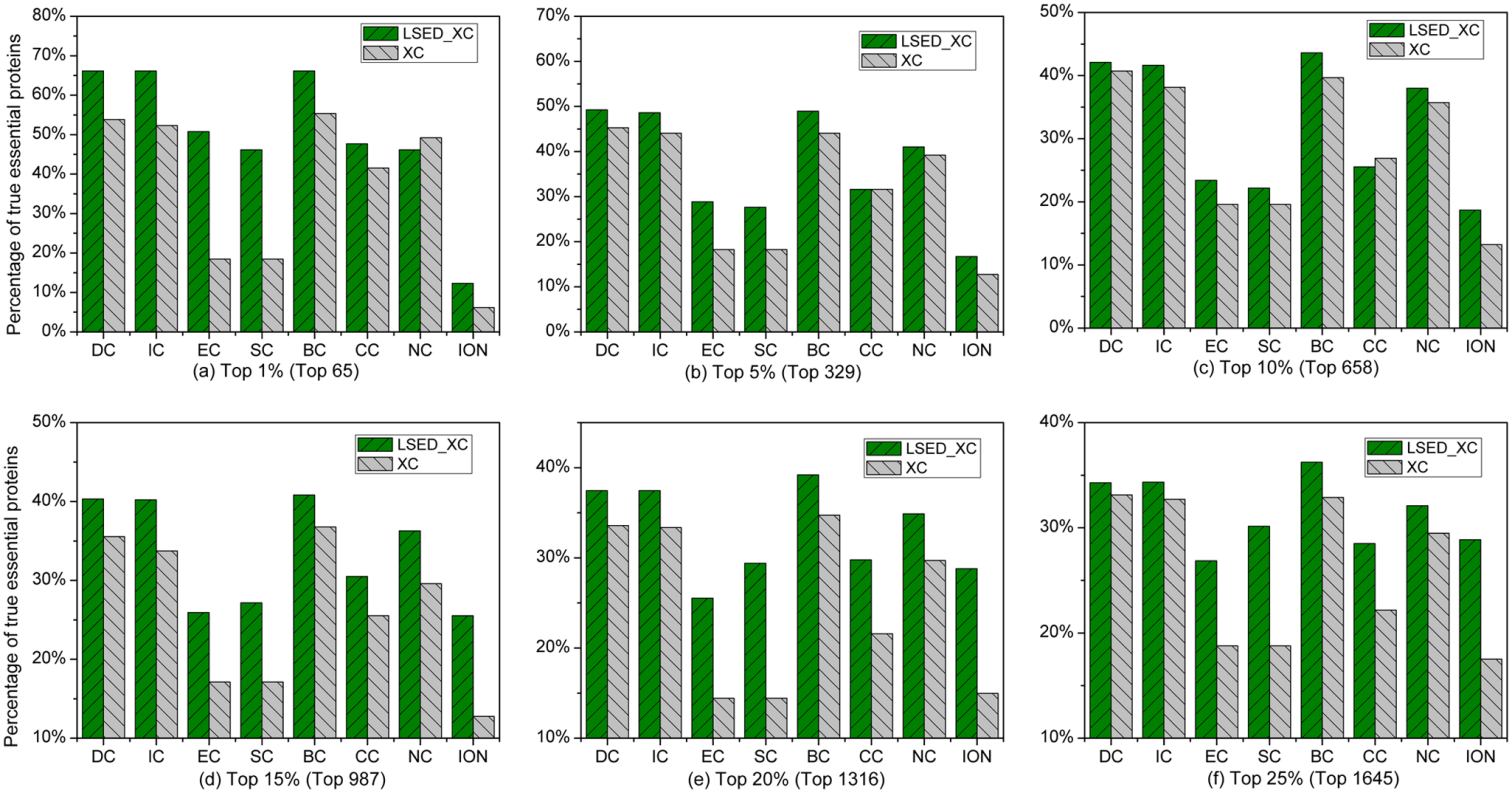
Percentage of top *c*% ranked proteins, identified by LSED-XC methods and XC methods, to be essential proteins of *Mus musculus*. Eight centrality methods (DC, BC, CC, SC, EC, IC, NC, and ION) were adopted to calculate centrality scores from the global PIN, respectively. LSED was combined with these centrality methods to calculate Localization-specific Centrality Scores from PSLINs separately. In (a)-(f), all the centrality methods are denoted as XC in the legend, and LSED with different XC methods are denoted as LSED-XC in the legend. The proteins are ranked in the descending order based on their Localization-specific Centrality Scores (*LCSs*) and centrality scores computed by LSED-XC methods and XC methods, respectively. Then, top 1%, 5%, 10%, 15%, 20% and 25% of the ranked proteins are selected as candidates for essential proteins. According to the list of known essential proteins, the percentages of true essential proteins were calculated. The figure shows the percentage of true essential proteins identified by each method in each top percentage of ranked proteins. The digits in brackets stand for the number of proteins ranked in each top percentage. For example, the total number of ranked proteins of *Mus musculus* is 6,582, thus the number of proteins ranked in top 1% is about 65 (= 6,582 *1%).

The percentages of true essential proteins of *Mus musculus* which are correctly predicted by LSED-XC methods are compared to those by XC methods in the top 1%–25% of ranked proteins, as shown in Fig 5. It is evident that the enrichment levels of true essential proteins identified by LSED-XC methods in each top percentage of ranked proteins are higher than those of the corresponding XC methods. DC, IC and BC outperform other XC methods for the top 1%–25% ranked proteins. From Table 4, we can find that, compared with DC, the *AIAcc* of LSED-DC in the top 1%–25% of ranked proteins is 10.5%. Compared with IC, the *AIAcc* of LSED-IC is 13.7% in the top 1%–25% of ranked proteins, and the *AIAcc* of LSED-BC is 12.4% in the top 1%–25% of ranked proteins comparing with BC.

As shown in Table 4, we can find that LSED also achieves improvements on the prediction accuracy of other centrality methods. Compared with EC, the *IAcc*s of LSED-EC are 175%, 58.3%, 19.3%, 51.4%, 76.8%, and 43% in the top 1%, 5%, 10%, 15%, 20%, and 25% of ranked proteins, respectively. Compared with SC, the *AIAcc* of LSED-SC is 77.25% in the top 1%–25% of ranked proteins. Compared with CC, the *AIAcc* of LSED-CC is 15.2% in the top 1%–25% of ranked proteins. In the top 1%–25% of ranked proteins, compared with NC, the *AIAcc* of LSED-NC is 9.9%. The percentages of true essential proteins correctly identified by ION in top 1%–20% ranked proteins are quite lower than those of topology-based centrality methods, while LSED-ION always identifies more true essential proteins than ION. For more information about the true essential proteins in the top percentages of proteins ranked by LSED-XC methods and XC methods in *Mus musculus*, see S4 Table in the Supporting Information files of this paper.

**Table 4:** The improved accuracy (*IAcc*) of LSED-XC method compared to the accuracy of the corresponding XC method in the top *c*% of ranked proteins of *Mus musculus*.

Method	*IAcc*					
	Top 1%	Top 5%	Top 10%	Top 15%	Top 20%	Top 25%
LSED-DC	22.86%	8.72%	3.36%	13.39%	11.54%	3.49%
LSED-IC	20.93%	9.38%	8.39%	16.12%	10.95%	4.78%
LSED-EC	175.00%	58.33%	19.38%	51.48%	76.84%	43.04%
LSED-SC	150.00%	51.67%	13.18%	58.58%	103.68%	60.52%
LSED-BC	19.44%	11.03%	9.96%	11.02%	12.91%	10.17%
LSED-CC	14.81%	0.00%	−5.08%	19.44%	38.03%	28.49%
LSED-NC	−6.25%	4.65%	6.38%	22.60%	17.39%	8.87%
LSED-ION	100.00%	30.95%	41.38%	100.00%	92.39%	64.93%

### Drosophila melanogaster

**Fig 6:**
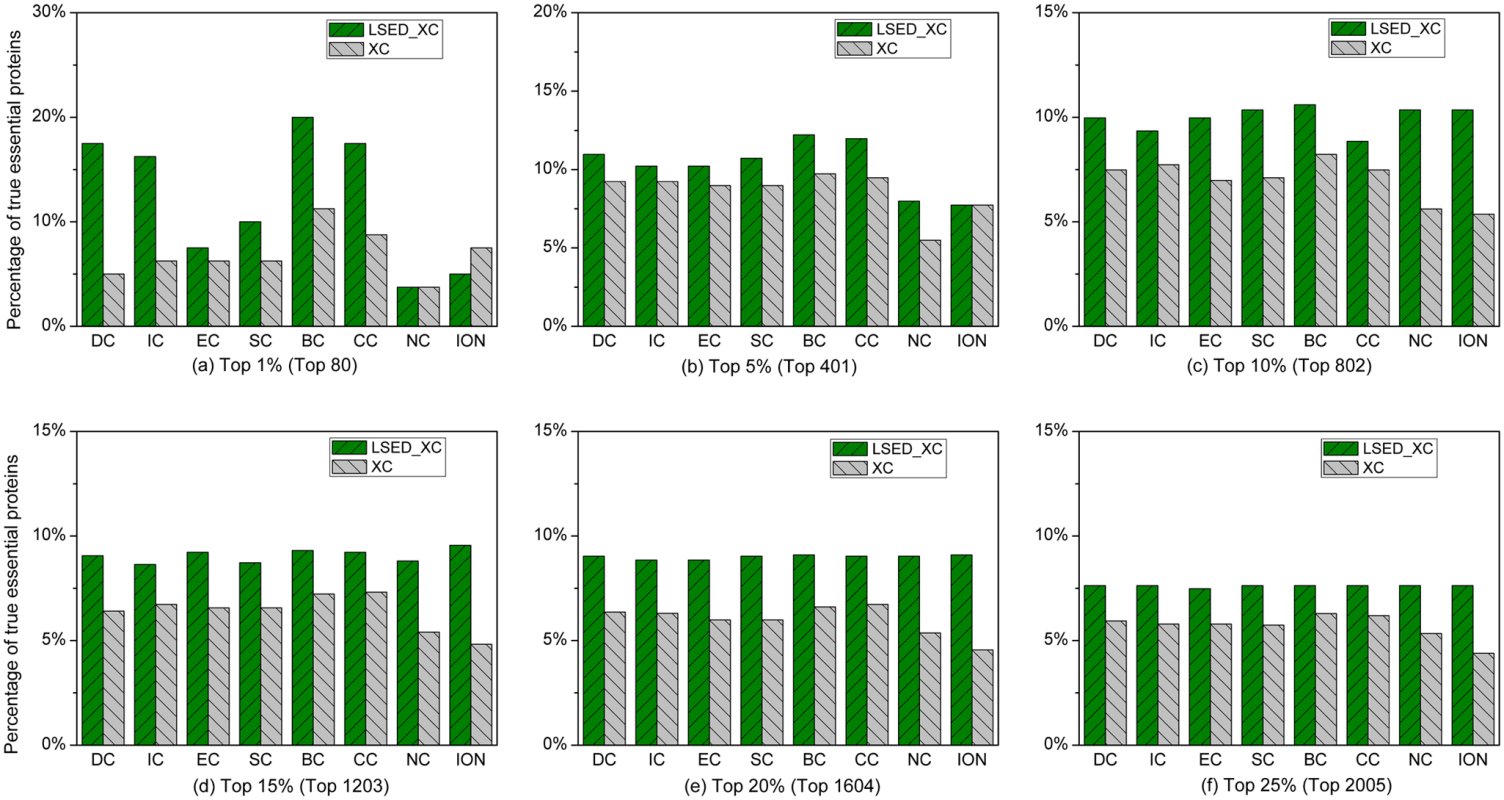
Percentage of top *c*% ranked proteins, identified by LSED-XC methods and XC methods, to be essential proteins of *Drosophila melanogaster*. Eight centrality methods (DC, BC, CC, SC, EC, IC, NC, and ION) were adopted to calculate centrality scores from the global PIN, respectively. LSED was combined with these centrality methods to calculate Localization-specific Centrality Scores from PSLINs separately. In (a)-(f), all the centrality methods are denoted as XC in the legend, and LSED with different XC methods are denoted as LSED-XC in the legend. The proteins are ranked in the descending order based on their Localization-specific Centrality Scores (*LCSs*) and centrality scores computed by LSED-XC methods and XC methods, respectively. Then, top 1%, 5%, 10%, 15%, 20% and 25% of the ranked proteins are selected as candidates for essential proteins. According to the list of known essential proteins, the percentages of true essential proteins were calculated. The figure shows the percentage of true essential proteins identified by each method in each top percentage of ranked proteins. The digits in brackets stand for the number of proteins ranked in each top percentage. For example, the total number of ranked proteins of *Drosophila melanogaster* is 8,020, thus the number of proteins ranked in top 1% is about 80 (= 8,020 *1%).

Fig 6 shows the percentages of true essential proteins of *Drosophila melanogaster* correctly predicted by LSED-XC methods and XC methods in the top 1%–25% of ranked proteins. Compared with XC methods,the improvements on the enrichment level of essential proteins obtained by LSED-XC methods can be observed in Fig 6 and Table 5. Specifically, LSED-BC identifies more true essential proteins than others in the top 1% to 10% of ranked proteins, more true essential proteins are correctly predicted by LSED-EC in the top 15% and 20% of ranked proteins, and the percentages of true essential proteins correctly predicted by most LSED-XC methods in the top 25% of ranked proteins are nearly the same, which are higher than those predicted by XC methods. For more information about the true essential proteins in the top percentages of proteins ranked by LSED-XC methods and XC methods in *Drosophila melanogaster*, see S5 Table in the Supporting Information files of this paper.

**Table 5:** The improved accuracy (*IAcc*) of LSED-XC method compared to the accuracy of the corresponding XC method in the top *c*% of ranked proteins of *Drosophila melanogaster*.

Method	*IAcc*					
	Top 1%	Top 5%	Top 10%	Top 15%	Top 20%	Top 25%
LSED-DC	250.00%	18.92%	33.33%	41.56%	42.16%	28.57%
LSED-IC	61.54%	9.76%	17.33%	22.12%	28.87%	24.18%
LSED-EC	20.00%	13.89%	42.86%	40.51%	47.92%	29.31%
LSED-SC	60.00%	19.44%	45.61%	32.91%	51.04%	33.04%
LSED-BC	77.78%	25.64%	28.79%	28.74%	37.74%	21.43%
LSED-CC	100.00%	26.32%	18.33%	26.14%	34.26%	23.39%
LSED-NC	0.00%	45.45%	84.44%	63.08%	68.60%	42.99%
LSED-ION	−33.33%	0.00%	93.02%	98.28%	100.00%	73.86%

### Average Accuracy over Species

From the comparison of the top percentages of ranked proteins for four species, it is clearly observed that some methods can work well for one species, but may fail for the other species. For example, ION can identify more true essential proteins than other methods for *Saccharomyces cerevisiae*, while the performance of ION is quite poor for other species. The performance of BC for *Saccharomyces cerevisiae* is not very good, but it outperforms other XC methods for other species, so does the LSED-BC method. A good essential protein prediction method should be not species-specific. Otherwise, it will be difficult for biologists to make a choice which method should be applied to a species without knowing the preference of the methods.

**Fig 7:**
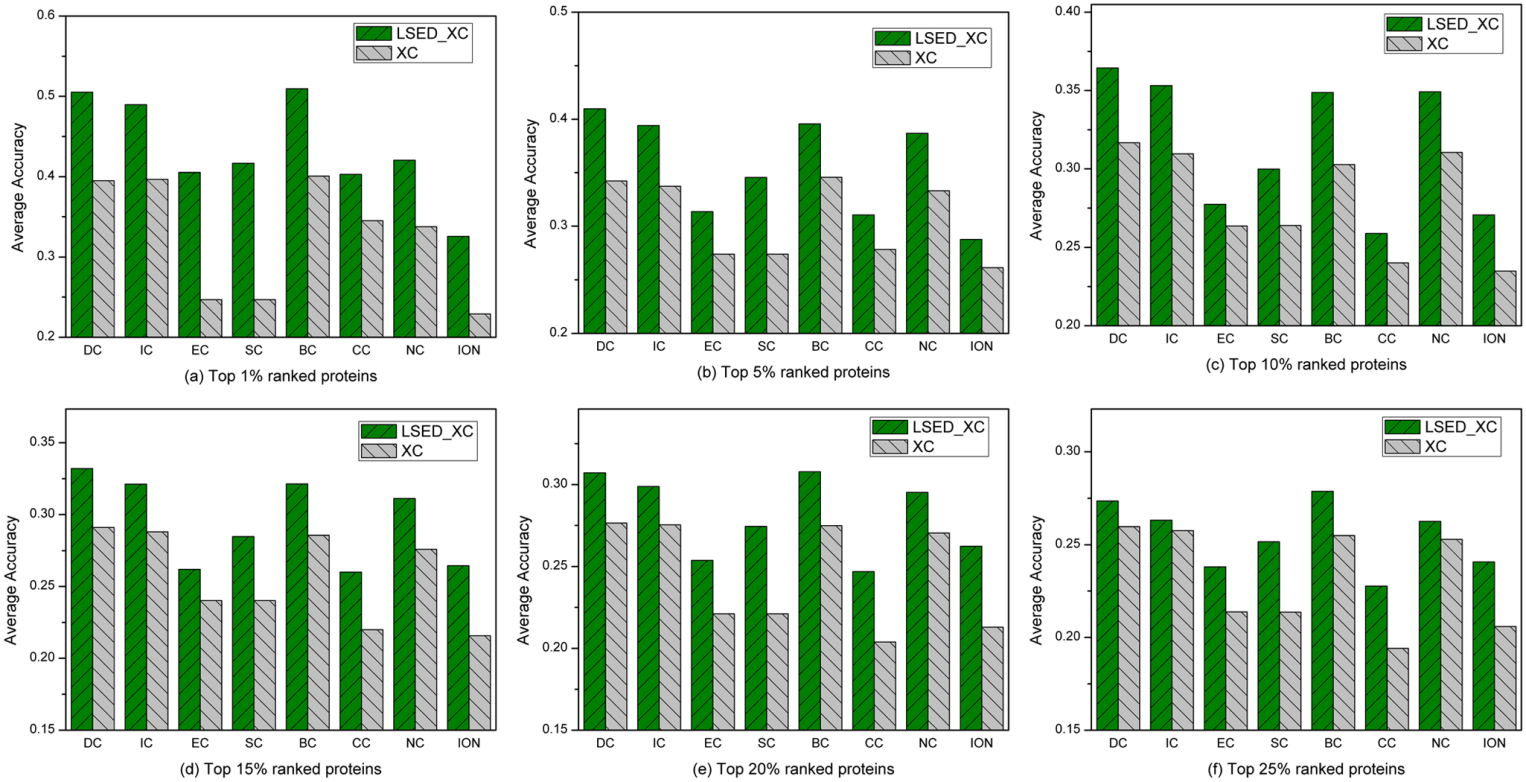
The *AKAcc*s of each method in different top percentages of ranked proteins over four species. Four species are *Saccharomyces cerevisiae*, *Homo sapiens*, *Mus musculus* and *Drosophila melanogaster*. When top 1%, 5%, 10%, 15%, 20% and 25% of the ranked proteins are selected as candidates for essential proteins, according to the list of known essential proteins of each species, the *Acc* of each method in each top percentage of ranked proteins was calculated. (a)-(f) illustrate the *AKAcc*s of LSED-XC methods and XC methods in the top 1%, 5%, 10%, 15%, 20%, and 25% of ranked proteins over four species, respectively. In (a)-(f), all the centrality methods are denoted as XC in the legend, and LSED with different XC methods are denoted as LSED-XC in the legend.

The *AKAcc* of each method in each top percentage of ranked proteins over four species was calculated. As shown in Fig 7, the *AKAcc*s of LSED-XC methods in each top percentage of ranked proteins are higher than those of XC methods consistently. Especially, the *AKAcc*s of LSED-BC and LSED-DC are always higher, compared with XC methods and other LSED-XC methods, which indicates their superior performances to identify essential proteins for different species. Furthermore, the higher *AKAcc*s of LSED-XC methods suggest that in most situations the *LCS*s taking into consideration the cellular compartments seem to be more predictive than the centrality scores measured in the global PINs and the essential proteins of different species can be explored better in the PSLINs.

## Discussion

Through the comparison, it is observed that LSED-XC can identify more true essential proteins than the corresponding XC method in the top 1%–25% of ranked proteins in most situations. Furthermore, LSED-XC methods gained higher *AKAcc*s in each top percentage of ranked proteins over four species, which indicates their better prediction performance for different species. In this section, we will look into the different predictions between the global PINs and the PSLINs, analyze the limitations of centrality methods applied to PSLINs, and discuss the confidence levels calculated in LSED.

### Different Predictions between the Global PINs and the PSLINs

**Fig 8:**
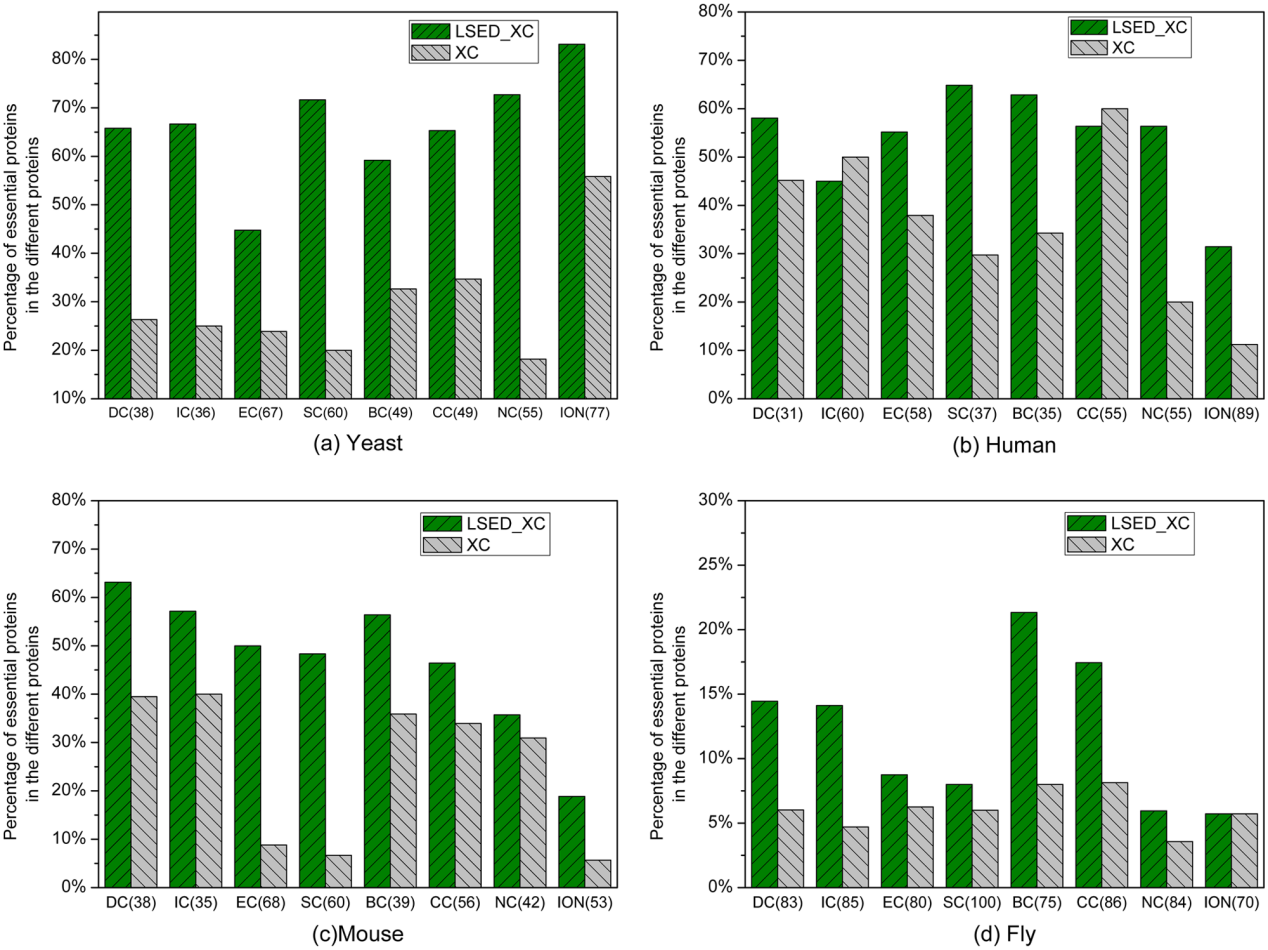
Percentage of different proteins, resulted by LSED-XC methods and the corresponding XC methods, to be essential proteins. Different proteins between two prediction methods are the proteins predicted by one method while neglected by the other method. (a)-(d) illustrate the percentages of true essential proteins in the different proteins from the top 100 protein sets ranked by LSED-XC methods and XC methods for *Saccharomyces cerevisiae*, *Homo sapiens*, *Mus musculus* and *Drosophila melanogaster*, respectively. In (a)-(d), the X axis represents the number of different proteins between LSED-XC and XC, and the Y axis represents the percentage of true essential proteins in the different proteins. In (a)-(d), all the centrality methods are denoted as XC in the legend, and LSED with different XC methods are denoted as LSED-XC in the legend.

LSED-XC methods measure the centrality of proteins based on the PSLINs, while XC methods measure the centrality of proteins based on the global PINs. To figure out the difference between essential proteins identified from the global PINs and the PSLINs, we compare the differences in the top 100 proteins ranked by XC methods and LSED-XC methods, respectively. In Fig 8, from (a) to (d), the X axis represents the number of different proteins between LSED-XC and XC, and the Y axis represents the percentage of true essential proteins in the different proteins. For example, DC(38) means that there are 38 different proteins in the two top 100 protein sets ranked by LSED-DC and DC, while there are 62 common proteins in the two top 100 ranked protein sets. In each different protein set, 65.7% ranked by LSED-DC are true essential proteins, while 26.3% ranked by DC are true essential proteins.

In Fig 8, in the top 100 proteins ranked by LSED-XC and its corresponding XC method, half or nearly half proteins are different. It seems that the PSLINs involved in LSED-XC and the strategy of calculating *LCS*s are the main reasons accounting for this difference. Compared with XC, the higher percentages of true essential proteins in the different proteins gained by LSED-XC demonstrate that the proteins with high *LCS*s measured from PSLINs are more likely to be essential. As shown in Fig 8(a), in the top 100 ranked proteins of *Saccharomyces cerevisiae*, LSED-XC methods find more true essential proteins than XC methods in those different proteins. For example, 40 out of 55 different proteins ranked by LSED-NC in top 100 ranked proteins are true essential proteins, while there are only 10 true essential proteins in 55 different proteins ranked by NC. In the top 100 ranked proteins of *Homo sapiens*, as illustrated in Fig 8(b), the average percentage of true essential proteins in the different proteins ranked by LSED-XC methods is 53.7%, while the average percentage of true essential proteins in the different proteins ranked by XC methods is 36%. In Fig 8(c), in the top 100 ranked proteins of *Mus musculus*, more true essential proteins are identified by LSED-XC methods than XC methods in those different proteins. About 47% of the different proteins ranked by LSED-XC methods are true essential proteins on average, while only 25% of the different proteins ranked by XC methods are true essential proteins. In Fig 8(d), we can observe that the top 100 proteins of *Drosophila melanogaster* ranked by LSED-XC methods and XC methods are quite different. Specifically, the top 100 proteins ranked by LSED-SC and SC are totally different, while the average number of different proteins ranked by other LSED-XC methods and XC methods is about 83. LSED-XC methods still find more true essential proteins than XC methods in those different proteins. Compared with the global PIN, the proteins with high *LCS*s measured from PSLINs are more likely to be essential. In another word, the centrality-lethality rule can be explained better in PSLINs.

### The Limitations of Centrality Methods Applied to PSLINs

In Figs 3, 4, and 6, we can observe that the percentages of true essential proteins identified by LSED with a centrality method, like CC, IC, and EC, are lower than those of the centrality methods applied to the global PINs in some situations. The reason might be the fact that these centrality methods are not applicable to networks with disconnected components [42, 43]. Compared with a global PIN, the PSLINs tend to be smaller and of less connectivity, containing more disconnected components. Closeness Centrality(CC) dysfunctions when the network contains disconnected components. The centrality scores of many proteins in PSLINs calculated by CC are 0, which have no power to measure the protein essentiality, while a few proteins will have centrality scores of 0 calculated by CC in the global PIN. Therefore, more proteins can be effectively ranked based on the global PIN, compared with PSLINs. IC is another closeness centrality method, similar to CC, facing the same problem [42]. Eigenvector Centrality(EC) is also not applicable to network with disconnected components [43]. Therefore, not every centrality method is proper for calculating the centrality scores of proteins in PSLINs, and more useful centrality methods are expected to be proposed for PSLINs with some disconnected components in the future.

### Different Reliability of Centrality Scores Calculated from Different PSLINs

The reliability of centrality scores calculated from different PSLINs are different. Take DC for example. As shown in Table 6, the number of true essential proteins in the top 100 proteins ranked by DC from each PSLIN of each species is compared. We can find out that the accuracies of these rankings from different PSLINs are different, and more essential proteins are ranked in top 100 proteins from networks with large sizes.

**Table 6:** The number of essential proteins in the top 100 proteins ranked by DC in the different PSLINs for four species.

PSLIN	# *Essential proteins*			
	*Saccharomyces cerevisiae*	*Homo sapiens*	*Mus musculus*	*Drosophila melanogaster*
*Cytoskeleton*	54	35	53	11
*Cytosol*	33	48	36	6
*Endoplasmic*	31	26	32	2
*Endosome*	13	34	33	1
*Extracellular*	0	50	43	11
*Golgi*	35	34	26	5
*Lysosome*(or *Vacuole*)	8	7	24	0
*Mitochondrion*	19	44	26	4
*Nucleus*	63	63	59	16
*Peroxisome*	1	1	15	0
*Plasma*	19	61	58	17

Subcellular localizations (Compartments) are used to denote different PSLINs.

#*Essential proteins* denotes the number of essential proteins in the top 100 proteins ranked by DC in a PSLIN of a species.

In LSED, the confidence levels of PSLINs are used to measure the reliability of centrality scores computed from different PSLINs, and the confidence level of a PSLIN is proportional to its size. The confidence levels of PSLINs are different, because different PSLINs play different roles and have different degrees of importance in cell activities. According to the incomplete statistics, as shown in Table 7, the numbers of proteins in PSLINs are not even, neither are the numbers of essential proteins. It is clearly observed that the number of essential proteins in PSLINs is roughly proportional to the number of proteins in the PSLINs. We can find that essential proteins are mainly distributed in the PSLIN of *Nucleus* for all four species. Many important cell activities, like chromosome replication and transcription, are carried in *Nucleus*, involving a large number of proteins. Therefore, the number of essential proteins in *Nucleus* PSLIN is larger than that of other PSLINs. Besides *Nucleus* PSLIN, the numbers of proteins in the PSLINs of *Plama*, *Cytoskeleton*, and *Cytosol* of *Homo sapiens*, *Mus musculus*, and *Drosophila melanogaster* are quite greater than those of other PSLINs, so are the numbers of essential proteins in these PSLINs. From the perspective of topology, the confidence levels of PSLINs are different, because different PSLINs have different destruction strengths in the global PIN. Highly connected proteins play an important role in maintaining the basic structure of a PSLIN, and the whole PSLIN will collapse if these proteins are removed. The collapse of a PSLIN with the larger size will contribute more to the destruction of the global PIN. Thus, in LSED, the confidence level of a PSLIN is proportional to its size.

**Table 7:** The distribution of proteins and essential proteins in the different PSLINs of four species.

PSLIN	*Saccharomyces cerevisiae*		*Homo sapiens*		*Mus musculus*		*Drosophila melanogaster*	
	#*P*	#*EP*	#*P*	#*EP*	#*P*	#*EP*	#*P*	#*EP*
*Cytoskeleton*	214	81	1302	318	653	161	283	30
*Cytosol*	349	94	2289	558	318	123	88	6
*Endoplasmic*	378	113	623	150	141	39	44	2
*Endosome*	118	14	453	101	85	34	5	1
*Extracellular*	13	0	856	206	151	77	106	12
*Golgi*	202	50	618	135	105	34	27	5
*Lysosome*(or *Vacuole*)	170	10	133	31	23	7	4	0
*Mitochondrion*	853	155	866	163	197	63	143	5
*Nucleus*	2110	698	5234	1205	2536	738	1028	90
*Peroxisome*	55	1	86	15	5	1	6	0
*Plasma*	343	45	2478	610	861	322	159	27

Subcellular localizations (Compartments) are used to denote different PSLINs. #*P* and #*EP* denote the numbers of proteins and essential proteins in a PSLIN of a species, respectively.

In conclusion, the centrality-lethality rule is rechecked in the scope of PSLINs by using LSED method which can be combined with a centrality method to identify essential proteins from PSLINs. Through the comparison on the prediction accuracy between LSED-XC with PSLINs and the corresponding centrality methods with the global PINs, we have found that proteins with high *LCS*s measured from PSLINs are more likely to be essential and the performance of centrality methods can be improved by LSED. From the biological angle, certain activities are carried out in each PSLIN, and the removal of proteins with high centrality scores will disturb these activities. As a result, the organism can not survive or grow. From the perspective of topology, proteins with high centrality scores play important roles in maintaining the basic structure of a PSLIN, and the whole PSLIN will collapse if these proteins are removed. Moreover, the collapse of PSLINs will lead to the collapse of the global PIN. Thus, the centrality-lethality rule can be supported better in the scope of PSLINs, and the essentiality of proteins can be more accurately predicted by *LCS*s measured from PSLINs.

## Supporting Information

S1 Dataset The PSLINs of *Saccharomyces cerevisiae*. It contains PSLINs of 11compartments, including *Endoplasmic*, *Cytoskeleton*, *Golgi*, *Cytosol*, *Vacuole*, *Mitochondrion*, *Endosome*, *Plasma*, *Nucleus*, *Peroxisome* and *Extracellular*.
(ZIP)

S2 Dataset The PSLINs of *Homo sapiens*. It contains PSLINs of 11compartments, including *Endoplasmic*, *Cytoskeleton*, *Golgi*, *Cytosol*, *Lysosome*, *Mitochondrion*, *Endosome*, *Plasma*, *Nucleus*, *Peroxisome* and *Extracellular*.
(ZIP)

S3 Dataset The PSLINs of *Mus musculus*. It contains PSLINs of 11compartments, including *Endoplasmic*, *Cytoskeleton*, *Golgi*, *Cytosol*, *Lysosome*, *Mitochondrion*, *Endosome*, *Plasma*, *Nucleus*, *Peroxisome* and *Extracellular*.
(ZIP)

S4 Dataset The PSLINs of *Drosophila melanogaster*. It contains PSLINs of 11compartments, including *Endoplasmic*, *Cytoskeleton*, *Golgi*, *Cytosol*, *Lysosome*, *Mitochondrion*, *Endosome*, *Plasma*, *Nucleus*, *Peroxisome* and *Extracellular*.
(ZIP)

S1 Table The confidence levels of different PSLINs of four species. (DOC)

S2 Table The true essential proteins in the top percentages of proteins ranked by LSED-XC methods and XC methods in *Saccharomyces cerevisiae*. (XLS)

S3 Table The true essential proteins in the top percentages of proteins ranked by LSED-XC methods and XC methods in *Homo sapiens*. (XLS)

S4 Table The true essential proteins in the top percentages of proteins ranked by LSED-XC methods and XC methods in *Mus musculus*. (XLS)

S5 Table The true essential proteins in the top percentages of proteins ranked by LSED-XC methods and XC methods in *Drosophila melanogaster*. (XLS)

S1 File The lists of *Saccharomyces cerevisiae* proteins with their centrality scores calculated by DC, BC, SC, EC, IC, CC, NC, PeC, as well as ION methods, and localization-specific centrality scores calculated by LSED-DC, LSED-BC, LSED-SC, LSED-EC, LSED-IC, LSED-CC, LSED-NC, LSED-PeC, as well as LSED-ION. (XLS)

S2 File The lists of *Homo sapiens* proteins with their centrality scores calculated by DC, BC, SC, EC, IC, CC, NC, as well as ION methods, and localization-specific centrality scores calculated by LSED-DC, LSED-BC, LSED-SC, LSED-EC, LSED-IC, LSED-CC, LSED-NC, as well as LSED-ION. (XLS)

S3 File The lists of *Mus musculus* proteins with their centrality scores calculated by DC, BC, SC, EC, IC, CC, NC, as well as ION methods, and localization-specific centrality scores calculated by LSED-DC, LSED-BC, LSED-SC, LSED-EC, LSED-IC, LSED-CC, LSED-NC, as well as LSED-ION. (XLS)

S4 File The lists of *Drosophila melanogaster* proteins with their centrality scores calculated by DC, BC, SC, EC, IC, CC, NC, as well as ION methods, and localization-specific centrality scores calculated by LSED-DC, LSED-BC, LSED-SC, LSED-EC, LSED-IC, LSED-CC, LSED-NC, as well as LSED-ION. (XLS)
