# Supplementary material for: Rechecking the Centrality-Lethality Rule in the Scope of Protein Subcellular Localization Interaction Networks
Source: PLoS One. 2015 Jun 26;10(6):e0130743. doi: 10.1371/journal.pone.0130743 (PMC4482623; doi:10.1371/journal.pone.0130743)
Supplement: S1 Table — (DOC) [file pone.0130743.s005.doc]

. **Table S1 The confidence levels of different PSLINs of four species**

| PSLIN | *Confidence Level* | | | |
| --- | --- | --- | --- | --- |
|  | *Y east* | *Human* | *Mouse* | *Fly* |
| *Cytoskeleton* | 0.101421800947867 | 0.248758119984715 | 0.257492113564669 | 0.275291828793774 |
| *Cytosol* | 0.165402843601896 | 0.437332823844096 | 0.125394321766562 | 0.0856031128404669 |
| *Endoplasmic* | 0.17914691943128 | 0.119029423003439 | 0.0555993690851735 | 0.0428015564202335 |
| *Endosome* | 0.0559241706161137 | 0.0865494841421475 | 0.0335173501577287 | 0.00486381322957198 |
| *Extracellular* | 0.00616113744075829 | 0.163546045089797 | 0.0595425867507886 | 0.103112840466926 |
| *Golgi* | 0.0957345971563981 | 0.118074130683989 | 0.041403785488959 | 0.0262645914396887 |
| *Lysosome*(or *Vacuole*) | 0.0805687203791469 | 0.0254107756973634 | 0.00906940063091483 | 0.00389105058365759 |
| *Mitochondrion* | 0.404265402843602 | 0.165456629728697 | 0.0776813880126183 | 0.139105058365759 |
| *Nucleus* | 1 | 1 | 1 | 1 |
| *Peroxisome* | 0.0260663507109005 | 0.0164310278945357 | 0.00197160883280757 | 0.00583657587548638 |
| *Plasma* | 0.162559241706161 | 0.473442873519297 | 0.339511041009464 | 0.154669260700389 |

Subcellular localizations (Compartments) are used to denote different PSLINs.
